# „Sport ist nicht austauschbar!“

**DOI:** 10.1007/s43594-023-00083-2

**Published:** 2023-04-25

**Authors:** Stefan Raid, Nils Neuber, Peter Lautenbach

**Affiliations:** 1 Deutsche Sportjugend, Hamburg, Deutschland; 2 Westfälische Wilhelms-Universität Münster, Münster, Deutschland; 3 Deutsche Sportjugend, Frankfurt am Main, Deutschland

## Liebe Leserinnen und Leser,

am 13. Dezember 2023 ist in der Berliner Max-Schmeling-Halle das „Signal zum Aufbruch“ erfolgt – hin zu einer Gesellschaft, in der ausreichende, tägliche Bewegung zum Alltag insbesondere für Kinder und Jugendliche werden soll. Bis allerdings die Forderungen nach „Bewegung und Sport für alle“, die der Bewegungsgipfel des Bundes, der Ländern, der Kommunen und des organisierten Sports formuliert hat, in der sportlichen Praxis ankommen, dürfte noch viel Wasser die Spree hinunterfließen. Als Herausgeber dieser Zeitschrift möchten wir daher noch einmal das zentrale Anliegen des Kinder- und Jugendsports adressieren: verlässliche Rahmenbedingungen für ein bewegtes Aufwachsen von Kindern und Jugendlichen.

Bewegungs‑, Spiel- und Sportangebote gehören zu den häufigsten und subjektiv wichtigsten Aktivitäten im Aufwachsen von Kindern und Jugendlichen. Die Bewegungsorte könnten dabei vielfältiger nicht sein – von organisierten Angeboten im Sportverein, im Ganztag oder in der Kinder- und Jugendhilfe über Sportunterricht in Schule oder Angebote in Kindertagesstätten bis hin zu Orten selbstorganisierten Sports oder kommerziellen Einrichtungen. Wo auch immer betrieben, haben Bewegung, Spiel und Sport ein hohes Erlebnispotenzial. Dabei können Bewegungsfreude, Anerkennung und soziale Zugehörigkeit erlebt werden und Freundschaften entstehen. Zugleich haben Bewegung, Spiel und Sport ein hohes Lernpotenzial: Nicht nur die motorische Leistungsfähigkeit kann sich verbessern, auch Selbstvertrauen, Sozialkompetenz, Selbstwirksamkeit und demokratische Partizipation können gelernt und erlebt werden. Darüber hinaus gilt der Zusammenhang zwischen Bewegung und kognitiven Lernprozessen seit langem als gesichert. Und schließlich ist die hohe gesundheitliche Bedeutung von Bewegung, Spiel und Sport vielfach belegt.

Insgesamt zeigt das: Sport ist nicht austauschbar! Es existiert kein anderes soziales Phänomen, das eine vergleichbare Bedeutung für das Aufwachsen von Kindern und Jugendlichen hat.

## Der passende Rahmen

Bei allem (Eigen‑)Lob wissen wir aber auch um die Herausforderungen: Der Sport ist kein Selbstläufer und auch kein Allheilmittel. Genauso wie Kinder und Jugendliche Anerkennung und Zugehörigkeit im Sport erleben, können sie dort Missachtung und Ausgrenzung erfahren. Es braucht also passende Rahmenbedingungen und angemessene Inszenierungen, damit Bewegung, Spiel und Sport ihr Potenzial entfalten können. Vor dem Hintergrund der gesellschaftlichen Veränderungsprozesse der vergangenen 10 bis 20 Jahre ist das allerdings nicht eben einfach.

Demografischer Wandel, soziale Ungleichheiten und neue Familienstrukturen wirken sich auf Kindheit und Jugend aus. Die Bildschirmzeiten nehmen zu, auch die Selbstoptimierung der „Generation Workout“ ist eine Herausforderung für den Kinder- und Jugendsport. Genauso erfordern strukturelle Veränderungen „Bewegung“ von Verbänden und Vereinen: Beispielsweise verbringen Kinder und Jugendliche durch die Ausweitung des öffentlichen Erziehungs- und Bildungsauftrags mit dem Ausbau von Ganztagskindertagesstäten und -schulen heute deutlich mehr Zeit in öffentlichen Einrichtungen als früher. Letztlich wissen wir aber wenig Systematisches über die Veränderungen des Kinder- und Jugendsports. Die letzte repräsentative Längsschnittstudie liegt 20 Jahre zurück.

Schließlich kam die Corona-Pandemie mit all ihren Auswirkungen hinzu: Bewegungsmangel, gesundheitliche Beeinträchtigungen, psychische Probleme, schulische Lernrückstände, Rückgang des Sportengagements, fehlende Teilhabemöglichkeiten, Abnahme sozialer Kontakte, Rückgang des jungen Ehrenamts und vieles weitere mehr. Gut deshalb, dass der Bewegungsgipfel Ende des vergangenen Jahres Einigkeit demonstriert hat, Bewegungs‑, Spiel- und Sportangebote für und mit jungen Menschen systematisch wieder auf- und auszubauen. Klar muss dabei stets sein, dass dies eine gesamtgesellschaftliche Aufgabe ist, die nur gemeinsam gelöst werden kann und von staatlichen Institutionen flankiert werden muss. Die Rahmenbedingungen müssen stimmen. Eine ressortübergreifende Zusammenarbeit auf Augenhöhe von Bund, Ländern, Gemeinden, Zivilgesellschaft, Wissenschaft und Praxis, wie sie beim Bewegungsgipfel angekündigt wurde, ist daher eine gute Grundlage, um bestehende Konzepte, Initiativen und Programme zu sichten, zu bündeln und auszubauen. Die Initiative, die Aktivitäten zum Kinder- und Jugendsport in dem im Koalitionsvertrag angekündigten Entwicklungsplan Sport zu bündeln, begrüßen wir daher ausdrücklich.

## Lösungsansätze und Forschung

Für ein solches bundesweites Programm zur Förderung des Kinder- und Jugendsports bieten sich folgende Ansatzpunkte an:

1. Eine frühkindliche Bewegungsförderung in Quartieren mit schwierigen sozialen Lagen unterstützt Integration, Sprache und Gesundheit. Zahlreiche Untersuchungen zeigen, dass frühe Interventionen nicht nur möglich, sondern mit Blick auf den weiteren Entwicklungsverlauf der Kinder auch sehr erfolgreich sind.
2. Mehr Bewegung in Kindertagesstätte und Schule können das Lernen und die individuelle Entwicklung begünstigen, unter anderem durch die Förderung von exekutiven Funktionen und Selbststeuerung. Langjährige Erfahrungen zeigen, dass leibliche Erfahrungen hoch lern- und entwicklungsbedeutsam sind.
3. Eine bessere kommunale Zusammenarbeit für mehr Bewegung im Ganztag, verbunden mit einer engen Kooperation von Schulen, Vereinen und weiteren Partnern, hilft bei der Bewältigung der Herausforderungen, die mit dem Rechtsanspruch auf einen Ganztagsplatz ab dem Jahr 2026 verbunden sind.

Ein letzter Punkt, der oben schon angeklungen ist: Guter Sport braucht gute Forschung. Ein breit angelegtes Forschungsprojekt kann die Datengrundlage für ein zielgerichtetes, strategisches Handeln im Kinder- und Jugendsport schaffen. Daher gilt: Auch in Sachen Forschung muss etwas passieren!

## Vielfältige Einblicke

Die vorliegende Ausgabe unserer Zeitschrift *Forum Kinder und Jugendsport *gibt wieder vielfältige Einblicke und Anregungen für Forschung und Praxis. Zu Beginn greift diesmal DOSB-Mitarbeiterin *Viola Kaets* in der Rubrik „Das aktuelle Stichwort“ das Thema „mentale Gesundheit“ auf. Im ersten Forschungsbeitrag analysieren *Sebastian Gehrmann, Laura Schreiner, Marlene Hansjürgens* und* Valerie Kastrup *dann das Sportengagement von Jugendlichen in Deutschland im Bereich der Individualsportarten. Sie untersuchen, ob sich ein Trend in Richtung Individualsportarten tatsächlich empirisch beobachten lässt und überprüfen, was sich dahinter verbirgt. Den bei *Forum Kinder- und Jugendsport* üblichen Dialog nehmen hierzu *sieben junge Sportlerinnen und Sportler* aus ganz unterschiedlichen Settings auf. Sie berichten, warum sie Individual- oder Mannschaftssport betreiben und warum sie dies entweder selbstorganisiert oder in einem Verein tun. In gewisser Weise greift auch der Fachbeitrag von *Melanie Hagikian, Julia Grob und Tim Bindel *dieses Feld auf. Sie untersuchen die Beweggründe von Jugendlichen für einen Verbleib in den Strukturen des Vereinssports.

Über unsere Dialogpartnerin im großen Interview freuen wir uns sehr: *Sabine Walper*, Direktorin und Vorstandsvorsitzende des Deutschen Jugendinstituts, beantwortet Fragen der Redaktion zur Rolle des Kinder- und Jugendsports „im Konzert“ der Jugendorganisationen und -verbände und zu Themen der Kinder- und Jugendsportforschung.

In einem weiteren Forschungsbeitrag analysieren *Julia Limmeroth, Lea Jebram, Florian Heussner, Norbert Hagemann *und* Volker Scheid* ein digitales Sportangebot für Grundschulkinder während der Covid-19-Pandemie und diskutieren mögliche Synergieeffekte hinsichtlich der Verknüpfung analoger und digitaler Formate. Zwei der am Projekt beteiligten Teamer*innen, *Julius Rücker *und *Yolanda Kappes Castro*, berichten im anschließenden Dialogbeitrag von ihren persönlichen Erfahrungen.

Zwei Fachbeiträge beleuchten in dieser Ausgabe Themen aus der Perspektive von Sportarten: *Marco Gößmann-Schmitt* vom Bayerischen Radsportverband skizziert aus seiner Perspektive als Landestrainer, wie der Drop-out im Kinder- und Jugendsport verhindert, eine positive Lernumgebung für Kinder und Jugendliche im Sport geschaffen und das Thema der pädagogische Trainingsqualität in der Erwachsenenbildung implementiert werden kann. Sportartbezogen ist auch die Projektvorstellung von *Malte Stoffers.* Darin geht es um die kognitive Förderung im Jugendfußball und wie hier die Förderung exekutiver Funktionen gelingen kann.

Der dritte Forschungsbeitrag in diesem Heft von *Esther Pürgstaller* behandelt das Körperverständnis von Heranwachsenden im Kontext der Mediatisierung und beschäftigt sich mit der Frage, inwiefern juvenile, digital-ästhetische Körperpraktiken auch für die bewegungsbezogene Vermittlung im Kinder- und Jugendsport bedeutsam sein können.

Um soziale Innovationen im Kinder- und Jugendsport geht es im Fachbeitrag von* Lukas Oettle.* Er möchte damit eine Debatte über die Bewältigung gesellschaftlicher Herausforderungen im und durch den organisierten Sport anstoßen.

Auch für Buchrezensionen bietet unsere Zeitschrift ein Forum. In dieser Ausgabe bespricht *Fabienne Bartsch* den Band „Weiblich – muslimisch – sportengagiert“ von Natalia Fast und nimmt damit sportbezogene Biografien türkeistämmiger Frauen in Deutschland in den Blick sowie die Handlungsempfehlungen der Autorin auch für den organisierten Sport.

Zu guter Letzt nutzen wir diese Ausgabe, um die beiden großen Forschungsverbünde zum Kinder- und Jugendsport vorzustellen und verbinden das mit Fragen an die Sprecher*innen *Jessica Süßenbach* (dsj-Forschungsverbund) und *Nils Neuber* (Forschungsverbund Kinder- und Jugendsport NRW). Schließlich kann es ohne adäquate begleitende Forschung keine positive Entwicklung des Kinder- und Jugendsports geben, davon sind wir überzeugt. In diesem Sinne wünschen wir Ihnen und Euch wieder viele neue Informationen und Anregungen durch die Lektüre!

Stefan Raid

(für die Institutionellen Herausgeber*innen)

Nils Neuber

(für die Herausgeber*innen der Forschungsbeiträge)

Peter Lautenbach

(für die Herausgeber*innen der Fachbeiträge)

